# Hospital Fire Incidents: Challenges and Solutions in a Developing Nation

**DOI:** 10.7759/cureus.80594

**Published:** 2025-03-14

**Authors:** Shamendra A Sahu, Jiten K Mishra, Abhijith Valsalan, Jalaz J Rahmi, Aparajita Saha

**Affiliations:** 1 Burns and Plastic Surgery, All India Institute of Medical Sciences, Raipur, Raipur, IND

**Keywords:** burn accidents, covid, fire hazard, fire hazards, fire incidents, hospital fire accidents, hospital fire incidents

## Abstract

During the COVID-19 pandemic, healthcare facilities across the globe were overwhelmed by the sudden influx of patients requiring urgent care, and hospitals had to rapidly adapt to this unprecedented crisis. In India, there was a sharp rise in burn disasters in COVID care centers and intensive care units (ICUs), where the mortality rate in these incidents was high. These tragedies were not isolated events but occurred frequently across different regions, underscoring the vulnerability of healthcare infrastructure to fire hazards. Despite the numerous fire accidents that took place in hospitals and ICUs, many of the underlying causes and deficiencies remain unaddressed. By analyzing the information in this study, we aim to identify the potential causes of these disasters and modifications that can be applied thereafter. Recognizing the need to address this issue, we have undertaken a comprehensive collection of data on major burn accidents in hospitals across India since the onset of the COVID pandemic. All the data were collected and arranged sequentially. The data were scrutinized for location, timing, mortality, number of people rescued, and cause of fire. These data provide valuable insights into the frequency, locations, and circumstances of these incidents. Comparative analysis of the data of the fire disasters in medical facilities of India with that of the developed countries reveals that fire hazards in medical institutions are largely preventable.

## Introduction

The COVID-19 pandemic created a sudden and massive burden on the healthcare system, especially critical care facilities. This resulted in the expansion of existing and new healthcare facilities. With this, there was an increase in the number of fire hazards in COVID care centers. In the beginning, general hospitals were converted into COVID care facilities to manage COVID patients by providing general care, isolation, and symptomatic treatment. Critically ill patients were shifted to centers having intensive care facilities. But, due to the overwhelming number of critical cases, the requirement for ICU beds increased exponentially. So, hospital general beds/hotels were converted to ICUs by arranging and installing various critical care instruments.

According to the WHO, the hospital's design and infrastructure should be structured in such a way as to sustain the burden of patients in disasters and emergencies. A study panel studying principals for large-scale disaster or pandemics suggests that a hospital should be able to expand its intensive care facility by 200% above its baseline capacity in 48 to 72 hours [1]. However, various safety measures in such a situation may be compromised. Fire safety norms for hospitals in India are as per the guidelines of the National Disaster Management Authority and regulations as per the National Building Code 2005. The National Accreditation Board for Hospitals & Healthcare Providers (NABH) is the regulatory body responsible for ensuring that hospitals adhere to fire safety norms and standards in India [2].

There is a lack of data on hospital fire hazards in our country, as there is no central registry system [3]. As per research, between 2010 and 2019, there were 33 fatal fire incidents with 131 fatalities in India during the past 10 years [4]. In this manuscript, we have systematically summarized and analyzed the major fire incidents across India after 2020. We have tried to find out the probable causes. Data from our study are compared with the fire hazards that happened in the hospitals of other developed countries, which may help improve fire safety.

## Materials and methods

Study design

This study is a systematic narrative review of fire incidents that happened in medical facilities and hospitals across India. Three years of data from March 2020 to 2023 were collected through a systematic online search. The Google search engine was used to find the newspaper article using the following keywords: hospital fire, COVID-19 fire, hospital fire accidents, hospital fire incidents, hospital fire hazards, medical facility fire, and hospital burn disasters.

Inclusion and exclusion criteria

News articles in publicly available sources published in English were identified. Fire incidents in healthcare settings in which there was high causality or major loss were included in the study. Reports published in regional languages were excluded from the review. The incidents from March 2020 before the first COVID outbreak in the country were excluded as already reported in one study [4].

Sample size

After a thorough search and review of the articles, 27 fire incidents were found suitable for our study, which occurred in healthcare facilities or makeshift COVID care facilities. The data from the incidents were tabularized systematically to identify and extract the data for the review.

Study measures and analysis

The variables that were analyzed and extracted were, from each report: location, type of facility (COVID vs. non-COVID, government vs. private hospital), casualties (number of deaths and number of victims rescued), possible cause of the fire (electrical short circuit, oxygen cylinder explosion, etc.), time of occurrence (day vs. night) and some specific problems reported (e.g., lack of emergency exits, delayed fire response) if reported. Multiple sources were cross-verified for each incident to ensure data reliability. Descriptive statistics were used to summarize the frequency and distribution of fire incidents.

## Results

A total of 27 major incidents occurred over three years following the COVID crisis (Table 1). Twenty incidents (74%) were reported from private hospitals and 7 (26 %) from government hospitals. Out of 27, about 50% (n=14) of incidents happened in COVID-19 designated hospitals. The most common place for hospital fires was the ICU (59.25%, n=16). A total of 112 deaths were reported in these 27 incidents. Few were reported in makeshift COVID care facilities. These were shopping malls, grounds, or hotels converted into temporary hospitals to accommodate the surging number of COVID cases. Out of all recorded fire incidents, 19 occurred in private hospitals, 7 in government hospitals, and 1 in a makeshift COVID care facility.

**Table 1:** Details of fire disasters over the past three years (2020-2023) NOC: no objection certificate;

S.No	Place	Date Time	Probable Reason	Place	Casualties	Covid/Non-Covid	Type of facility/Remarks
1.	Welfare Hospital. Bharuch. Gujarat [5]	01.05.2021 12:35 am	Short circuit	Covid Ward	18 deaths (16 covid patients, 2 nurses)	COVID Hospital	Private; Fire broke out in the new building with no fire NOC
2.	Prime Criticare Hospital. Kausa-Mumbra Thane [6]	28.04.2021 3:40 am	Short circuit in the meter box	Meter room in the ground floor	4 deaths. 16 rescued.	Non-COVID patients	Private hospital failed to submit a fire safety audit report
3.	Ayush Hospital. Surat [7]	25.04.2021 11.40 pm	Blast in AC due to short circuit or overloading	COVID ICU	No death 16 rescued.	COVID patients	Private
4.	Vijay Vallabh Hospital. Virar. Mumbai [8]	23.04.2021 3:00 am	Central AC sparks and blast in ICU	COVID ICU	15 deaths.	COVID care hospital	Private. No sprinkler system
5.	Rajdhani Hospital. Raipur. Chhattisgarh [9]	17.04. 2021 5:00 pm	Short circuit in the fan	COVID ICU	5 deaths. 29 rescued.	COVID care hospital	Private. Fire extinguishers were not used on time
6.	Well Treat Hospital Nagpur [10]	9.04.2021 8:10 pm	AC unit	ICU	4 deaths.	Non-COVID	Private. The building is not sanctioned and lacks a fire NOC and system. The hospital was operational despite the closure notice issued by the municipal
7.	Makeshift facility (Jumbo COVID center). Dahisar. Mumbai [11]	04.04.21 12:00 pm	Short circuit in the electrical wiring.	-	No deaths. 50 rescued.	COVID	Private. Fire doused by the fire extinguisher at the center
8.	Patidar Hospital & Research Center. Ujjain [12]	04/04/2021 11.30 am	Short circuit in the power board of the ICU	ICU	4 injured, 21 shifted	COVID	Private. Immediately after the fire broke out, the hospital staff shut off the electricity and broke the window panes
9.	Safdarjung Hospital. New Delhi [13]	31/03/2021 6:15 am	Short circuit in the ventilator due to voltage fluctuations.	ICU	50 rescued	Non-COVID	Government. Regular fire drills conducted in the hospital
10	Sunrise Hospital in Dreams Mall at Bhandup West. Mumbai [14]	26.03.2021 12:30 am	Not known	Not known	11 deaths 70 Rescued	COVID	Makeshift private hospital at mall
11.	Institute of Cardiology and Cardiac Surgery. Kanpur [15]	28.03.2021 7:30 am	Short circuit leading to a fire in a storeroom on the ground floor near the ICU.	Storeroom	175 rescued	Non-COVID	Government. A fire extinguisher was used to douse the flames
12.	Shree Vijay Vallabh Sarvajanik Hospital. Vadodara [16]	17.03.2021 9:00 pm	Not Known	Not Known	23 rescued, including 17 COVID-19 patients	COVID and Non-COVID	Private. Only one staircase in the hospital
13.	Sun Hospital. Cuttack [17]	1.02.2021	Not known	Not given	11 patients rescued	Non-COVID	Private. The approach road to the hospital was narrow, creating hurdles for firefighters. No fire safety license. Construction work was going on in the top floor
14.	District General Hospital. Bhandara [18]	09.01.2021 2:00 am	Short circuit in the electric warmer.	SNCU	10 deaths (infants) 7 rescued	Non-COVID	Government
15.	Uday Shivanand Covid Hospital. Rajkot [19]	27.11.2020 12:30 am	Not given	ICU	5 deaths due to asphyxiation	COVID	The emergency exit in the ICU was blocked. There was no adequate ventilation system in the unit. The hospital has only one entry and exit (only one-meter wide). Staff were not trained in firefighting. The hospital didn’t have any emergency evacuation plan or automatic sprinkler system
16.	Little Flower Hospital, Ahmedabad, Gujarat [20]	09.12.2020 Afternoon	Oxygen supply pipe	Not known	No death	COVID	Private
17.	Apex Hospital, Mulund [21]	3.10.2020 Late night	Overheating of a generator set	ICU	1 death, 40 shifted	Non-COVID	Private
18.	Chhatrapati Pramila Raje Civil Hospital, Kolhapur [22]	28.09.2020 3.25 am	Electrical short circuit	Trauma unit	No death	COVID	Government
19.	Sadguru Covid Hospital, Jagatpur, Cuttack [23]	21.09.2020 12.45 pm	AC short circuit	ICU	127 rescued	COVID + Non-COVID	Private. Sprinklers worked on time. No fire safety certificate
20.	Vadodara Municipal Corporation-run SSG Hospital [24]	8.09.2020 7:00 pm	Short circuit in the ventilator and the ICU	ICU	No death, 39 rescued	COVID	Government. Fire doused with an extinguisher
21.	Guru Gobind Singh Hospital, Jamnagar, Gujarat [25]	25.08.2020	Short circuit	Old ICU	No death, 9 non-COVID patients rescued	Non-COVID	Government
22.	Hotel Swarna Palace, Hotel converted as COVID quarantine, Vijayawada [26]	09.8.2020 5 am	Short circuit in AC	Reception lobby	10 deaths, 22 injured, 32 rescued	COVID	Private
23.	Shrey Hospital, Navrangpura, Ahmedabad [27]	06.08.2020 3.30 am	Short circuit	ICU	8 deaths, 40 rescued	COVID	Private
24.	New Life Multispecialty Hospital in Chandal Bhata of Gohalpur, Jabalpur, Madhya Pradesh [28]	01.08.2022 3:00 pm	Generator short circuit	-	8 deaths, 13 injured	Non-COVID	Private. Absence of emergency fire exits and inadequate fire extinguishers
25.	Civil Hospital Ahmednagar, Maharashtra [29]	06.11.2021 11:00 am	Short circuit	ICU	11 deaths	COVID	A total of 17 patients were present in the ICU, out of which 11 died of asphyxia, absence of a sprinkler system.
26.	Rohini's Brahm Shakti Hospital, New Delhi [30]	11.06.2022 5:00 am	Short circuit	ICU	1 death	Non-COVID	Private
27.	Honey Children's Hospital, Shihori, Gujarat [31]	15.03.2023 5.45 am	Short circuit	Neonatal ICU	1 death (newborn), 2 children rescued	Non-COVID	Private

fIn almost all incidents reported, electrical appliances and short circuits were the cause of fire in our analysis. A short circuit in the wiring system is the most common cause of fire. Air conditioners, ventilators, warmers, generators, meter boxes, and fans are the other causes of a fire starting (Table 2). One reported cause of the fire was a malfunction in the oxygen supply line.

**Table 2:** Various causes of fire accidents in hospitals in India

Cause of Fire	Number of events
Short Circuit	9
Air Conditioner	5
Not Known	4
Ventilator	2
Generator	2
Meter Box	2
Fan	1
Warmer	1
Oxygen Pipeline	1

**Figure 1:**
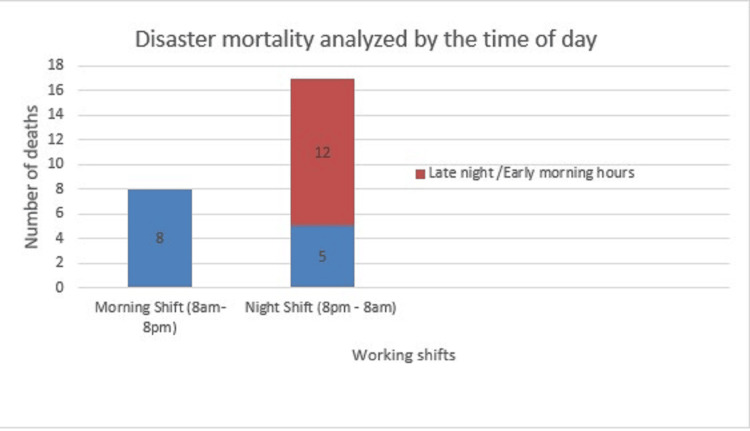
Disaster mortality analyzed by the time of day

Of the total fire disasters, 65.38% (n=17) occurred during the night shift (8 pm-8 am), out of which 14 (84.35%) occurred during late night/early morning hours (12 am-6 am). Only 8 of 27 incidents happened during the morning shift (8 am-8 pm) (Figure 1).

## Discussion

Disaster preparedness and policy-making require a detailed assessment of the problem. First, developing countries lack a database of burn injuries, and the need for an audit is felt only after a disaster. No collective database is maintained for burn disasters in hospitals, housing complexes, commercial buildings, and various institutions [3]. Collection and analysis of the data help identify the problem and policy-making at the level of a service provider like hospitals/medical establishments.

In India, the process of policymaking and enforcement cascades from central authorities to state-level bodies, including the National Disaster Management Authority (NDMA), State Disaster Management Authorities (SDMAs), Regional Disaster Management Centres (RDMCs), the National Accreditation Board for Hospitals & Healthcare Providers (NABH), the National Building Code (NBC), and state firefighting departments. The involvement of numerous regulatory and monitoring agencies weakens the system, making it difficult to effectively implement fire safety protocols. As a result, hospitals often find ways to flaunt fire safety protocols and regulations [32].

Building and maintaining a hospital as per the fire safety norms is the most important way to avoid fire hazards. But, with the rapid urbanization and expansion of healthcare facilities, hospitals have overlooked fire safety. As per an audit of one of the states of India, 90% of the hospitals do not obtain an NOC from the fire department, including private and government institutions [33]. So, policy-making, reporting, and enforcement should have better coordination for formulating and enforcing rules. The NOC, once obtained, should be renewed and reviewed regularly for better compliance with fire safety protocols. Following a fire incident, reporting and data collection can be done through an online portal to establish a central registry.

A study conducted in 2019 found that India recorded 33 major hospital fire incidents between 2010 and 2019, resulting in the loss of 131 patients. In contrast, 27 major hospital fire incidents occurred within just 3 years from 2020, leading to 112 reported deaths and 770 patients rescued [4]. This steep rise in burn disasters is due to overburdened ICUs and makeshift hospitals [34].

The analysis of factors causing fire hazards is the narrative of a hospital's standard of fire safety. An audit of fire incidents across the hospitals in the Latin American and Caribbean region and around the world revealed common causes: cooking equipment (52%), trash and debris (9%), electrical wiring and lighting (7%), heating equipment (4%), washing machines and driers (4%), electronic and medical equipment (4%), and smoking materials (2%) in decreasing order [35]. In another set of analyses published by the United States Fire Administration for 2012-2014, faulty electrical systems and appliances were the cause of life-threatening fire accidents in only 6% of cases [36]. This contrasts with the cause of fire disasters in India. According to a study by Choudhury in 2013, out of 22 major fire accidents in India, 21 (95%) were due to electrical short circuits, electricity, and electronics [37]. Pathalra SSR et al. also established that 78% of significant hospital fires broke out due to short circuits. According to that study, other non-electrical causes of fire were chemical overheating, such as spirit, the storage of flammable materials in basements, and the use of combustible materials during building renovations [4]. Our analysis of fire accidents in hospitals in India during and after COVID-19 also reveals that in 80% of cases, the cause of hospital fire is electrical appliances and wiring malfunction. Our analysis shows that the reason for most of the fire disasters in India is correctable and such disasters can be avoided.

Analysis of the equipment is essential to prevent such hazards. In our country, short circuits in electrical lines and air conditioners are the most common reasons to start a fire in a hospital (Table 2), whereas in developed countries, most fire hazards are related to cooking equipment [35].

Air conditioners are a common culprit in starting hospital fires. A window air conditioner is unsuitable for an ICU, as there are no air changes. Hospitals either use a split air conditioner or air handling unit (AHU). Ideally, hospitals should install AHUs for air handling. But, due to space and monetary constraints, AHU is not used in small hospital setups. In a split air conditioner, an indoor unit is usually installed near the patient's bed. The air conditioner ducting system contains multiple soldering points, along with electrical wiring and plug outlets situated close to the patient's bed. The area around the patient's bed has a high concentration of oxygen, particularly in the ICU, due to the continuous administration of oxygen. Even minor sparks in these circuits, typically harmless, can quickly ignite a fire in an oxygen-rich environment [37]. Fires caused by air conditioner units are often triggered by wiring short circuits, frequent voltage fluctuations, and the buildup of dirt, dust, and debris in the air filter and external unit. These factors lead to overheating, which can spark a fire, especially in an oxygen-rich environment [38]. To prevent fires caused by air conditioner units, consider implementing the following precautions: keep the indoor unit, wiring, and electrical panels away from the patient's bed; use a stabilizer to manage frequent voltage fluctuations; have the air conditioner professionally maintained and serviced annually; avoid using extension cords for the air conditioner; and ensure that flammable materials such as paper, gasoline, and debris are kept at least three feet away from the air conditioning units.

Ventilators are also commonly implicated as a cause of these types of disasters. Continuously running equipment can overheat and, when used with oxygen, may easily ignite a fire. The risk is further heightened if there is a leak of 100% oxygen, as it lowers the ignition threshold. To mitigate this risk, ventilators, heaters, oxygen concentrators, and other ICU machines operating continuously should be positioned 4-5 meters away from the patient's bed. In a fully operational ICU, monitoring the run time of each ventilator is advisable. Equipment with the highest run time should be prioritized for shutdown or standby. If an ICU bed becomes vacant, a patient on a ventilator with the longest run time can be transferred to the empty bed and the ventilator switched off or placed on standby. Additionally, heavy equipment like ventilators should ideally be equipped with an inbuilt warning system to alert when the machine overheats.

Since most fire breakouts were due to an electrical short circuit and electrical appliances, an electrical vigilance team should be formed to inspect the continuously running heavy instruments. Daily audits and charts should be made for the integrity of electrical circuits and overheating/overworked equipment. The overworked devices are to be taken care of on a priority basis as per daily audit.

A common factor contributing to increased fire outbreaks in hospitals during COVID times is the elevated use of oxygen due to the influx of critically ill patients. While oxygen itself is not flammable, its concentration can rise to 23.5% in enclosed spaces like intensive care units where 100% oxygen is used continuously, creating an "oxygen-rich" environment. In such conditions, even minor friction, a scratch, or a spark can ignite a fire [37].

Analysis of the timing of the occurrence of the incidents is equally essential. In the United States, hospital fire accidents commonly occur in the afternoon between noon and 1 pm as the most common cause was cooking, which relates to the timing it stated [36]. On the contrary, 65% of fire disasters in India occurred in night shifts, of which 82% happened in late night/early morning hours (12 pm - 8 am). A scientific explanation for this is increased oxygen-enriched air inside a closed space like an ICU during night hours. This is due to less movement and fewer door-opening incidences inside critical care units like ICUs. This issue may also arise from having fewer hospital staff on the night shift, coupled with the natural tendency for attention to wane during nighttime hours. To address this, simple adaptations can be implemented like sufficient staff being available during night shifts to maintain focus and vigilance, to provide ongoing training to night shift personnel to keep safety practices at the forefront, and to use monitoring systems to oversee critical areas. These measures can help mitigate risks and improve safety during nighttime hours.

Among the reported incidents in our study, 16 occurred in the ICU and 1 in a ward while the location in 6 incidents was not given. Other affected areas included the trauma unit, reception lobby, storeroom, and meter room. This highlights the high vulnerability of intensive care units to such incidents.

Many makeshift facilities have been used to treat patients during the COVID-19 pandemic. However, there are no standardized norms or criteria for establishing and maintaining these temporary healthcare facilities, which can lead to accidents. Makeshift hospitals are often set up to handle epidemics or disasters such as storms, earthquakes, and floods. Therefore, developing guidelines for these temporary facilities is essential to prevent mishaps. The absence of proper planning and standards has contributed to fire hazards and other safety issues. For example, recently, a fire broke out in a COVID makeshift facility due to a short circuit in a furniture shop having flammable materials located in the basement of the building [39].

In our analysis, we found certain factors that reduced the mortality in these incidents: using a fire extinguisher immediately [11], hospital staff cutting electricity and breaking windows [12], and a properly functioning firefighting system [23]. This shows that conducting regular fire drills helps to tackle such situations [13].

Similarly, some lacunas were identified in analyzing these fire disasters. Many issues were related to architectural deficiencies, such as having only one staircase in the hospital [16], a narrow approach road that hindered firefighters [17], a single entry and exit point, narrow entry and exit routes, lack of an emergency evacuation plan or automatic sprinkler system, and a blocked emergency exit in the ICU [19]. Few technical lapses were also in these disasters, like no sprinkler system [8,25], inadequate ventilation system [19] and Inadequate/absent fire extinguishers [24]. Improving and addressing these factors may help prevent or reduce the impact of such fire disasters.

This analysis has some limitations. Data from a few incidents of fire disasters may have been missed due to words used in the search engine. As there is no central registry system regarding fire disasters in the country, the data were taken from the paper article, so some details regarding the disaster may be absent.

## Conclusions

Fire hazards in hospitals are a major concern, yet most are preventable. Despite regulations and safety laws, the frequency of fire incidents is rising. To address this growing issue, there is a need for a national registry, enhanced enforcement mechanisms, improved collaboration between central and state authorities, and operational upgrades in intensive care units and wards. Makeshift hospitals and ICUs will remain vital in handling future emergencies. Thus, it is crucial to introduce administrative and operational reforms to prevent future fire disasters in healthcare facilities.
